# Modification of Whey Protein Isolate with Surfactants Based on Hofmeister Series and Interaction Parameter

**DOI:** 10.1021/acsomega.5c10293

**Published:** 2026-02-27

**Authors:** Jhenifer Stefani Lopes, Marina Fernandes Cosate de Andrade, Ana Rita Morales

**Affiliations:** Universidade Estadual de Campinas (UNICAMP), School of Chemical Engineering (FEQ), Department of Materials and Bioprocess Engineering (DEMBio), 28132 Cidade Universitária, Av. Albert Einstein 500 13083-852 Campinas, São Paulo, Brazil

## Abstract

Developing thermoplastic materials from proteins requires structural reorganization and stabilization of specific intermolecular interactions. In this study, we modified whey protein isolate (WPI) with different surfactants—two cationic (cetylpyridinium chloride, CPC, and benzalkonium chloride, BC) and one anionic (sodium dodecyl sulfate, SDS)—to evaluate their effects on the system’s structure and thermal and rheological properties. The Hofmeister series and interaction parameters between the components were taken into consideration. Characterization was achieved by Fourier transform infrared spectroscopy (FTIR), circular dichroism (CD), dynamic light scattering (DLS), thermogravimetric analysis (TGA), differential scanning calorimetry (DSC), and parallel plate rheometry. Results indicated changes induced by surfactants in the secondary conformation of proteins, particularly, the conversion of α-helical structures into β-sheets. Theoretical solubility analysis (Flory–Huggins model) predicted the miscibility for all surfactants. TGA found a greater reduction in the thermal stability. Rheological analyses showed a predominance of the elastic modulus (*G*′) over the viscous modulus (*G*″) even after thermal denaturation. Results were interpreted based on the expected interactions between the surfactants and the protein’s amino acids. Our findings suggest that modifying WPI with surfactants can be an effective strategy to tailor its structural and mechanical properties in a surfactant-dependent manner, provided that a suitable balance between protein denaturation, molecular reorganization, and thermal stability is maintained.

## Graphical abstract

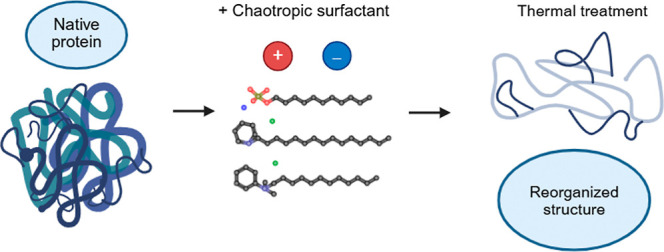

## Introduction

Increasing demand for sustainable solutions has driven research into using proteins as a source for producing thermoplastic materials. Whey protein isolate (WPI) has gained attention in this context, particularly in the packaging industry, due to its outstanding oxygen and water vapor barrier properties.^1–3^ These attributes make WPI a promising alternative to conventional petroleum-based plastics, aligning with contemporary environmental goals.^4–6^

Whey proteins are globular molecules with a significant portion of their structure in the form of α-helices. Amino acids are evenly distributed across their polypeptide chains in a balanced manner, encompassing acidic–basic and hydrophobic/hydrophilic regions. Primary proteins found in whey include β-lactoglobulin (β-LG), α-lactalbumin (α-LA), immunoglobulins (IGs), bovine serum albumin (BSA), bovine lactoferrin (BLF), and lactoperoxidase (LP), along with several minor components.^3,6^

Whey undergoes various treatments, generating products with distinct protein profiles such as whey protein concentrates (WPCs), which contain 35%, 50%, 65%, or 80% (w/w) protein, depending on the concentration. Products with a protein content exceeding 90% (w/w) are classified as whey protein isolate (WPI), a high-purity concentrate. Both WPCs and WPIs are used as carriers to enhance biological properties when incorporated into food products. Upon thermal treatment, α-LA denatures readily and can be separated by precipitation.^3,6,7^

WPI performance can be optimized by adding surfactants.^8–12^ These compounds can alter the physicochemical properties of proteins, facilitating the formation of complexes that enhance both the processability and functionality of the resulting materials, and it is essential to investigate how WPI and surfactants interact. Modifying WPI with surfactants can not only improve its barrier properties against gases and moisture but also enhance its mechanical properties, thus broadening its potential applications.^3,13^

Interactions between surfactants and proteins are complex and depend on the specific nature of each component. Surfactants can induce conformational changes in proteins, and the extent of these changes is governed by the surfactant concentration and structure. The apolar tails of surfactants interact with hydrophobic amino acid side chains, while polar and ionic groups can modify the protein structure. Depending on concentration, surfactants may significantly alter protein behavior, promoting new conformational states that affect the dynamics and functions of active sites as well as mechanisms of denaturation.^9,11,13–15^ Interaction between soluble proteins and surfactants often results in unfolding, with structural changes ranging from minor conformational shifts to substantial modifications in secondary and tertiary structure.^14,16^

Studies have focused on the interactions between cationic and anionic surfactants and proteins, especially the interactions involving charged head groups.^16–18^ Each surfactant interacts differently depending on its specific properties and organization of molecules, whether as isolated units in solution or in more complex arrangements like micelles. Critical micelle concentration (CMC) and ionic strength of the medium are also important factors to be considered.^18–20^

Interactions on globular proteins such as Bovine Serum Albumin (BSA) and surfactants like sodium dodecyl sulfate (SDS) have highlighted the complexity of these interactions that vary with surfactant concentration and involve different binding modes, directly impacting protein structure and function, including conformational changes and exposure of active sites.^20,21^ At low concentrations, SDS interacts with the hydrophobic residues of β-Lg inducing mild structural perturbations, whereas higher concentrations promote micelle formation resulting in more pronounced denaturation.^22,23^ Conversely, cationic surfactants such as cetylpyridinium chloride (CPC) primarily interact via electrostatic attractions with negatively charged residues on the protein surface. At neutral or alkaline pH, such interactions may form neutral complexes or precipitates, whereas at acidic pH—in which the protein carries a net positive charge—cationic surfactants contribute to maintaining protein solubility and preventing precipitation.^23^

Mixtures of hydrophobic proteins with water in the presence of surfactants can promote self-organization of these substances into various structures from modification of the aqueous system’s interfacial properties.^24–26^ The thermodynamics of these mixtures is governed by interactions between molecules of different phases and can be described by the Flory–Huggins interaction parameter (χ) which is commonly used to characterize interactions in polymer systems.^27–29^

While originally developed for synthetic polymers, the Flory–Huggins theory has been extended to describe food systems composed of polysaccharides, proteins, and low-molecular-weight sugars.^28,29^ Its application to systems involving surfactants, such as WPI–surfactant systems, is particularly relevant for understanding the interactions between proteins and surfactants in food systems.^30,31^

Hofmeister series describes the influence of ions on the physicochemical properties of proteins, impacting key processes such as aggregation and phase transitions^32,33^ Within this framework, salt ions are categorized as either kosmotropic (structure-stabilizing) or chaotropic (structure-destabilizing). Kosmotropic ions enhance protein stability by reinforcing structural integrity, whereas chaotropic ions promote destabilization by disrupting protein–water interactions.^13^ Recent studies indicate that the Hofmeister effect, driven by ionic interactions, significantly impacts protein microstructure and functionality.^34,35^

Typically, kosmotropic ions (on the left side of the series) increase surface tension and reduce protein solubility, producing a “salting-out” effect that enhances structural stability and prevents protein inactivation. Conversely, chaotropic ions (on the right side of the series) promote protein unfolding and increase solubility.^35^ Kosmotropic ions, characterized by their small ionic radii and strong hydration capacity, act as structure-forming agents, whereas chaotropic ions, with lower charge density and weaker hydration interactions, exhibit the opposite effect.^36,37^ The interplay between charge density and water interaction capacity in modulating these effects remains an area of growing interest in experimental research.

Considering the Hofmeister series and the Flory–Huggins Interaction Parameter of the components, this study investigated, as a novelty, the effects of two cationic ammonium surfactants—cetylpyridinium chloride (CPC) and benzalkonium chloride (BC)—and one anionic surfactant, sodium dodecyl sulfate (SDS), on WIP thermal processing. The objective was to evaluate how addition of surfactant before and after thermal protein denaturation modulates electrostatic interactions and affects the rheological properties of the resulting samples.

## Materials

The material used included Whey Protein Isolate (WPI) with a minimum protein content of 90% on a dry weight basis, Hilmar 9010 Instantized Whey Protein Isolate (Hilmar Ingredients) with a minimum 90% protein on a dry basis (USA), sodium sulfite (Synth) analytical standard purity 99% (CHN), and the surfactants: cetylpyridinium chloride (CPC) (Sigma Chemical Co.) analytical standard purity 99% (DEU), benzalkonium chloride (BC) (Sigma Chemical Co.) analytical standard purity 95% (DEU), and sodium dodecyl sulfate (SDS) (Dinâmica Co.) analytical standard purity 99% (IN).

## Methods

### WPI Modification

The miscibility of WPI with surfactants was predicted by initially determining the solubility parameter using the methodology described by Krevelen and Nijenhuis (2009).^27^ The total solubility parameter (δt) and its individual components—dispersive (δd), polar (δp), and hydrogen bonding (δh)—were calculated for both WPI and surfactants. Additionally, the Flory–Huggins interaction parameter (χ) was evaluated. To enrich the discussion, the results were contrasted with the theoretical expectations derived from the Hofmeister series, which encompasses the perspective on the effect of charged species released into the aqueous medium.

Commercial WPI characterization used SDS-PAGE electrophoresis under nonreducing conditions to identify the primary protein fractions, performed in triplicate. Samples were vortexed and dissolved in a buffer containing 20 mM Tris/HCl, 5 mM EDTA, 2.5% SDS, and 5.0% 2-mercaptoethanol at pH 8.0. The samples were then heated in boiling water for 2 min. Bromophenol blue was added at a final concentration of approximately 0.1%. Sample concentration was adjusted to 2 mg/mL. Gel electrophoresis was performed following Farrell (1998).^38^

Before heat treatment, the WPI–surfactant systems were expected to undergo structural changes due to specific interactions between the protein and each surfactant, potentially affecting the solubility, stability, and aggregate size. Heat treatment was applied to further unfold the proteins, amplifying these interactions and allowing better assessment of their effects on aggregate formation and dispersion behavior.^39–41^

### Preparation of WPI and WPI–Surfactant Formulations

WPI modification was performed according to Lopes et al. (2023)^42^ and the formulations are shown in Table 1.

**Table 1:** Formulations of WPI and Surfactans

Formulation	WPI	WPI + SDS	WPI + CPC	WPI + BC
Deionized water (g)	100	100	100	100
WPI (g)	10	10	10	10
Sodium sulfite (g)	0.2	0.2	0.2	0.2
SDS (g)	0	10	0	0
CPC (g)	0	0	10	0
BC (g)	0	0	0	10

All solid reagents were first manually homogenized. Next, water was added to the system. Once a uniform mixture was formed, the resulting suspension was processed by using a Shaker Marconi MA 259 mechanical shaker at 510 rpm for 30 min. After being shaken, the mixture was subsequently subjected to ultrasonic treatment in an ultrasonic bath for 15 min to ensure complete dispersion.

### Viscosity Measurements before Thermal Treatment

Viscosity of the WPI + surfactant samples in solution was measured before the thermal denaturation process using a Brookfield DV3T viscometer equipped with Spindle 18 at a controlled temperature of 25 °C. A working range was established with the torque when starting the spindle rotation in the sample, reaching a value of at least 10% and at most should not exceed a torque of 90% throughout the analysis. As WPI + BC had high viscosity, we had to adopt different spindle speed conditions to maintain the appropriate shear rate for reading.

### Thermal Denaturation, Drying, and Milling

The homogenized mixtures of each formulation described in Table 1 were transferred into molds and heated at 90 °C for 30 min to induce protein denaturation. Subsequently, the materials were dried in a vacuum oven at 40 °C and 21 mmHg for 48 h. After drying, the samples were ground using a Freezer/Mill 6870 cryogenic mill under the following conditions: five precooling cycles in liquid nitrogen (5 min each), an operating time of 2 min, an intermediate cooling step of 1 min in liquid nitrogen, and a milling rate of 10 Hz. Lyophilization was carried out after freezing the samples in an ultrafreezer; the frozen materials were then dried for 72 h in a freeze-dryer (model L101, Liobras). All samples after the denaturation heat treatment were considered as thermoplastic proteins, and were named WPIT without surfactants and WPIT + SDS, WPIT + CPC, and WPIT + BC with surfactants.

The thermoplastic samples, after treatment and freeze-drying, were characterized by Fourier Transform Infrared Spectroscopy (FTIR), Circular Dichroism (CD), Differential Scanning Calorimetry (DSC), Thermogravimetric analyzer (TGA), and rheological analysis.

### Dynamic Light Scattering (DLS)

Dynamic Light Scattering (DLS) was employed to evaluate the effect of surfactants on the colloidal behavior and hydrodynamic size of the whey protein isolate (WPI). Measurements were performed by using a Malvern Zetasizer Nano ZS (Malvern Panalytical). The formulations prepared for the modified proteins (Table 1) were not suitable for DLS due to their high solids content, which caused multiple scattering and prevented reliable correlation function fitting. Therefore, a distinct sample preparation protocol was adopted exclusively for DLS, following and adapting the procedure described by Eissa (2019).^43^

### Sample Preparation DLS

The samples were prepared in phosphate buffer by adjusting the initial WPI concentration to 5% (w/w). Surfactant-containing formulations were prepared at two concentrations, 0.1% and 1% (w/w), according to Table 2, which reports the exact masses of WPI and each surfactant added per 100 mL of buffer. All formulations were heated at 90 °C for 30 min to induce controlled protein denaturation and ensure comparable initial conditions among the samples.

**Table 2:** Formulations Prepared for DLS Analysis (Prepared in 100 mL of Phosphate Buffer).

Formulation	WPI (g)	SDS (g)	CPC (g)	BC (g)
WPI	5.0	–	–	–
WPI + SDS 0.1%	5.0	0.10	–	–
WPI + SDS 1%	5.0	1.00	–	–
WPI + CPC 0.1%	5.0	–	0.10	–
WPI + CPC 1%	5.0	–	1.00	–
WPI + BC 0.1%	5.0	–	–	0.10
WPI + BC 1%	5.0	–	–	1.00

Prior to analysis, all samples were diluted 1:20 (v/v) in phosphate buffer to achieve the optimal scattering intensity required for DLS measurements and to prevent detector saturation and multiple scattering effects. After dilution, the final concentrations were 0.05% (w/v) protein and either 0.005% or 0.05% (w/v) surfactant, depending on the initial formulation. Measurements were performed in triplicate using disposable cuvettes appropriate for diluted dispersions. Results are reported as the average ±standard deviation. This dilution step may disrupt large or weakly bound aggregates, potentially leading to size distributions that do not fully represent the structures present under concentrated conditions. However, DLS measurements require samples to fall within a specific scattering intensity range to ensure a reliable autocorrelation fitting. At higher concentrations, our samples exhibited pronounced multiple scattering and frequent detector saturation. Therefore, the applied dilution was necessary and is consistent with established practices for protein–surfactant systems (e.g., Eissa, 2019).^43^ Consequently, the reported DLS sizes should be interpreted as reflecting the dispersed state under diluted conditions rather than the native bulk formulation.

### Thermoplastic Samples: Structural and Thermal Characterization

Sample characterization used Fourier transform infrared (FTIR) spectroscopy using a Thermo Scientific Nicolet 6700 FT-IR Spectrometer (Madison, USA). Measurements were performed in transmittance mode using the SNAP-IN BASEPLATE accessory (KBr method) within the 4000–600 cm^–1^ range, with a resolution of 4 cm^–1^ and 32 scans per spectrum.

Circular dichroism (CD) spectroscopy analyzed the secondary structure of native and modified WPIT. Protein solutions were prepared by dissolving the powders of previously prepared thermoplastic samples (Table 1) in 0.01 M phosphate buffer (PBS) solution at pH 7.0, all to a final concentration of 0.2 mg/mL. CD spectra were recorded using a CD spectrometer with a 0.1 cm quartz cell at 20 °C, with a scan of 190 to 250 at 50 nm/min and a bandwidth of 1 nm. The buffer was used as a blank, and the data were expressed as the average ellipticity per residue.

Protein thermal transitions were observed using a TA Instruments Trios DSC2A-01974 differential scanning calorimeter (DSC), with cooling from 25 to −80 °C, followed by heating up to 200 °C. Heating and cooling rates were set to 10 °C/min, and both steps were performed under a nitrogen atmosphere. Sample thermal decomposition and stability were assessed by using a TA Instruments Trios 0550-1238 thermogravimetric analyzer (TGA) scanning from room temperature to 600 °C at a rate of 10 °C/min under a nitrogen atmosphere.

Rheological properties were evaluated by using a TA Instruments DHR 2 rheometer with parallel plate geometry. The distance between the plates was set at 1 mm. The measurements were performed using freeze-dried, denatured solid powders, which, upon heating to 160 °C, softened and exhibited melt-like behavior, behaving as an amorphous thermoplastic system, thus enabling reliable oscillatory rheological measurements without the need for prior compression molding or solvent-based processing. A strain sweep was performed at a frequency of 1.0 Hz and 160 °C to determine the appropriate deformation within the linear viscoelastic regime, and a strain amplitude of 0.1% was selected. The result of this analysis is provided as Figure S1 in the Supporting Information and shows that both storage (*G*′) and loss (*G*″) moduli remain practically constant up to approximately 0.1% strain, indicating a well-defined linear viscoelastic region (LVR). Dynamic frequency sweep measurements were then carried out over the angular frequency range of 0.01 to 500 rad/s at 160 °C.

## Results and Discussion

### Whey Protein Isolate Electrophoresis Characterization

WPI proteins were composed primarily of β-lactoglobulin (∼50–60%), α-lactalbumin (∼15–25%), and small amounts of immunoglobulins, lactoferrin, and glycomacropeptide.^44,45^ These components contain a variety of amino acids that may interact with the proposed surfactants. Electrophoretic analysis confirmed the presence of the main protein fractions by passing an electric current through a gel containing the molecules of interest. Based on their size and charge, the molecules move through the gel at different rates or in different directions, allowing their separation.^38,46,47^

Figure 1 shows the result of WPI electrophoresis, which revealed a strong band corresponding to β-LG dimers, indicating a high prevalence of the dimeric form. The prominent presence of dimers is likely attributable to the intrinsic propensity of the WPI sample for β-LG self-association and β-LG/α-LA coaggregation.^48,49^

**Figure 1:**
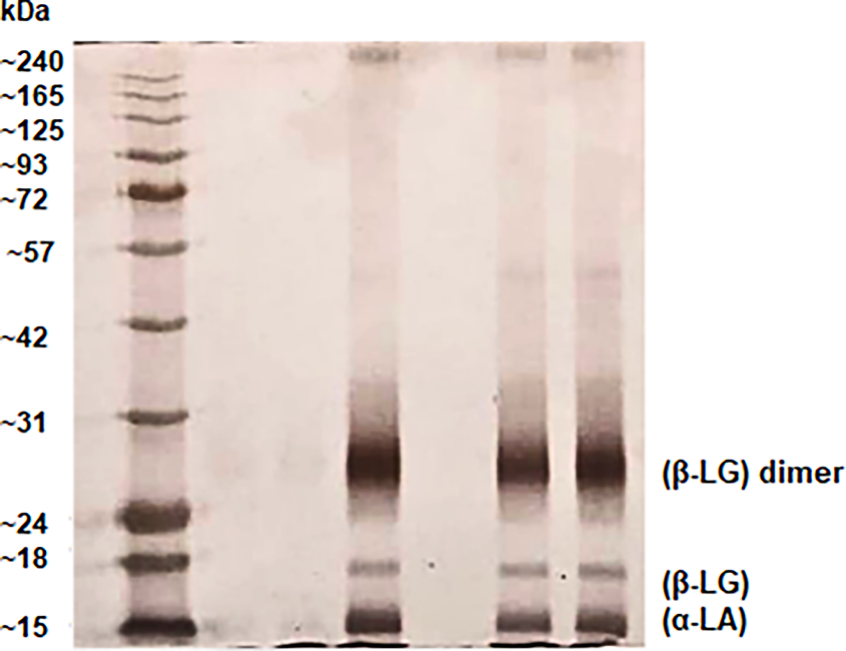
WPI Electrophoresis, (β-LG) β-lactoglobulin, (α-LA) α-lactalbumin.

β-Lactoglobulin (β-LG) is the primary protein component of WPI under native physiological pH conditions. It predominantly exists as a dimer but can dissociate into native monomers under either acidic or basic conditions. The monomeric form of β-LG is a globular protein with a subunit molecular weight of approximately 18 kDa. It contains two disulfide bonds (Cys66–Cys160 and Cys106–Cys119) and a free sulfhydryl (−SH) group at Cys121, which is believed to play a critical role in disulfide exchange reactions with other whey proteins or casein.^50^

Conversely, α-lactalbumin (α-LA) is a monomeric globular protein with a subunit molecular weight of approximately 14 kDa. It contains four intramolecular disulfide bonds, which confer greater thermal stability compared with β-LG and its two disulfide bridges.^50^

Moreover, the absence of additional bands—even after sample dilution for SDS-PAGE—suggests a high degree of purity in the WPI used. The results were consistent and showed repeatability, closely resembling the patterns found in the literature for native WPI.^49^

### Miscibility Prediction by the Flory–Huggins Interaction Parameter

Tables 3 and 4 show the total solubility parameters (δt) and those for dispersive (δd), polar (δp), and hydrogen bonding (δh) components of WPI and the Flory–Huggins interaction parameter (χ), respectively. For χ values <1, the mixture was characterized as miscible^27,51^ as expected for all systems. The smaller the difference between WPI δ values and the surfactant, the more compatible they are, which leads to a lower χ and greater protein solubility in the medium.^27,52^

**Table 3:** Solubility Parameters of Dispersive (δd), Polar (δp), Hydrogen-Bonding (δh) Components, and Total Solubility (δt).

Sample	δd (MPa)^05^	δp (MPa)^05^	δh (MPa)^05^	δt (MPa)^05^
WPI	17.00	5.80	14.90	23.30
SDS	16.69	6.31	6.88	19.13
CPC	17.00	4.78	5.13	18.39
BC	21.99	3.51	5.78	23.01

**Table 4:** Flory–Huggins Interaction Parameters (χ).

Interaction	Fllory–Huggins parameter (χ)
WPI + SDS	0.6198
WPI + CPC	0.8567
WPI + BC	0.0037

All evaluated surfactants, particularly benzalkonium chloride (BC), produced predictions suggesting potentially favorable interactions with whey protein isolate (WPI), as indicated by the lowest estimated interaction parameter values (Table 4). However, due to the complexity of surfactant structures and relevant molecular interactions, these results demand cautious interpretation. According to the Flory–Huggins model, the interaction parameter (χ) reflects both the component affinity and the presence of specific functional group interactions. While low χ values indicate thermodynamic miscibility, a comprehensive understanding also requires an analysis of surfactant stereochemistry relative to the amino acids in WPI.

The methodology based on Hildebrand and Hansen’s solubility parameters, frequently used in conjunction with the Flory–Huggins theory to estimate the interaction parameter, offers a powerful and easily applicable tool for initial screening and prediction of polymer–solvent miscibility.^27^ Its importance lies in its ability to simplify the thermodynamics of the mixture from the properties of functional groups, allowing for a quick and efficient estimation of the compatibility. However, this approach faces significant limitations as it is predominantly based on the theory of regular solutions, which fails to account for the nature and intensity of specific interactions.

Given these limitations, we contrasted our results with the Hofmeister series, which underscores the key practical shortcomings of the regular solution-based model.

We also consider that empirically, the Hofmeister series theory approach suggests the possibility that solubilization in aqueous systems with ions does not follow a general rule of polarity but rather the specific order of the hydration strength of the ions (such as the difference between sulfate, which precipitates, and iodide, which solubilizes).^53–55^

Therefore, if the Flory–Huggins parameters present overestimated values due to their limitations, the Hofmeister series perfectly illustrates that for systems involving charged species or strong directional interactions, conventional solubility parameters are insufficient, as they cannot predict the complexity of ion–solute–solvent interactions that dictate the actual behavior of the mixture.

Figure 2 presents the Hofmeister series as a conceptual and hypothetical framework to explore the possible positioning of the surfactant ions investigated and their expected qualitative effects on the protein behavior. Owing to their bulky molecular structures, long hydrophobic chains, and quaternary ammonium groups, the cationic surfactants CPC and BC are hypothetically located toward the chaotropic (protein-structure-disrupting) end of the cationic series. However, their large size and amphiphilic nature may lead to deviations from the classical Hofmeister trends established for small inorganic ions.

**Figure 2:**
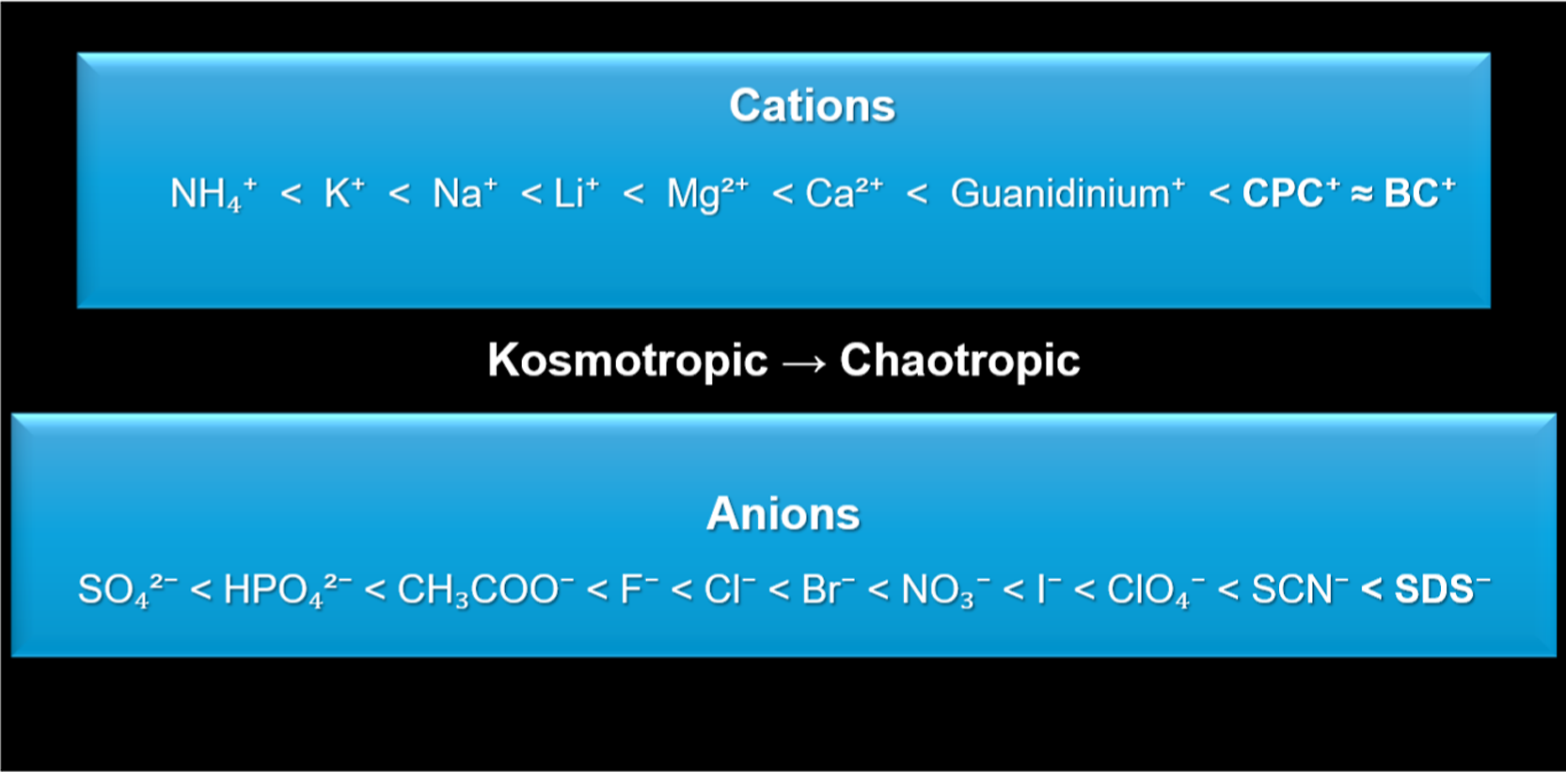
Hypothetical position of selected surfactants on the Hofmeister series. Adapted from ref 54 under the ACS AuthorChoice License. Copyright © 2020 American Chemical Society.

Similarly, SDS, as an anionic surfactant, is conceptually positioned closer to chaotropic anions, which are commonly associated with protein destabilization. It is essential to note that this representation does not aim to provide a definitive ranking but rather serves as a qualitative tool to guide the interpretation of ion–protein interactions within these complex surfactant–protein systems.

Electrophoresis analysis (Figure 1) confirmed that the WPI used is composed mainly of β-lactoglobulin and α-lactalbumin, proteins rich in diverse amino acids—including charged (Glu, Asp, Lys, Arg), polar (Ser, Thr, Gln), and aromatic (Phe, Tyr, Trp) residues. These chemical groups offer multiple sites for interaction with surfactant headgroups and tails, and the expected effects of these interactions are shown in Table 5.

**Table 5:** Expected Effects of Surfactant–WPI Interactions Considering the Amino Acids^14,56,58^

Surfactant (ion type)	Hofmeister classification	Interaction mechanisms^a^	Effect on protein unfolding	Effect on aggregation
**SDS (O** ^**–**^ **)**	Anionic/strong chaotrope	Strong electrostatic attraction to basic residues: **Lys (9.3%)** and **Arg (2%)**. Hydrophobic binding to nonpolar residues: **Leu (9.7%)**, **Ile (6.4%)**, **Val (5.2%)**, **Ala (4.9%)**. Formation of protein–surfactant micelles stabilizing unfolded conformations.	**Strong denaturant**: disrupts intramolecular interactions and exposes hydrophobic domains →**complete unfolding**.	**Dual behavior**: inhibits aggregation at optimal ratios (via chain shielding)but may promote aggregation under surfactant saturation.
**CPC (N** ^**+**^ **)**	Cationic/intermediate chaotrope	Electrostatic attraction to acidic residues: **Glu (16.2%)** and **Asp (10.2%)**. Hydrophobic interaction via long **C16 aliphatic chain**. π–π stacking with aromatic residues: **Phe (2.9%)**, **Tyr (2.7%)**, **Trp (1.9%)**.	**Moderate denaturant**: induces local unfolding through charge neutralization and hydrophobic collapse.	**Promotes aggregation**: reduces electrostatic repulsion and facilitates molecular association.
**BC (N** ^**+**^ **)**	Cationic/intermediate chaotrope	Similar electrostatic interactions with **Glu** and **Asp**. Hydrophobic interactions via shorter C10–C12 **aliphatic chains** (shorter than CPC). Benzyl group enables π–π stacking with aromatic residues.	**Moderate denaturant**: similar effect to CPC but **milder due to shorter hydrophobic tail**.	**Promotes aggregation**: same mechanism as CPC but with slightly lower intensity.

a The amino acid content, represented in g/100g of product, was obtained from the data sheet of the whey protein isolate used in this study, Hilmar 9010 Instantized Whey Protein Isolate (Hilmar Ingredients).

As a strong chaotropic anion, SDS shows a high capacity for disrupting the protein structure. Even in the absence of heat, it interacts primarily with basic residues (Lys, Arg, and His), solubilizes aggregates, and unfolds proteins via strong electrostatic and hydrophobic interactions. Although SDS can inhibit aggregation at subsaturation levels by shielding protein surfaces, at higher concentrations, it may induce aggregation via micelle clustering and charge inversion.^56–58^ CPC, a cationic surfactant, exhibits a potent interaction with acidic residues (Glu, Asp), and its long C16 chain promotes hydrophobic interaction with nonpolar regions exposed during partial unfolding. Its aromatic pyridinium ring adds π–π stacking capacity with residues like Trp and Phe, contributing to structural reorganization. These multivalent interactions can facilitate more ordered unfolding and refolding pathways compared with SDS.^15,59^ BC behaves similarly to CPC but possesses a shorter alkyl chain (C10–C12) and a benzyl group rather than a pyridinium ring, resulting in slightly reduced hydrophobic and π–π interaction potential. Nonetheless, BC also promotes aggregation and destabilization of native protein conformations, albeit to a lesser extent than CPC.^14,58^

When considering Hofmeister effects, SDS stands out due to its sulfate group which lies among the most chaotropic anions, facilitating protein unfolding and aggregation via salting-out mechanisms. Conversely, CPC and BC associated with Cl^–^ fall closer to the intermediate range of the Hofmeister scale, exerting less aggressive destabilizing effects, thus allowing more tunable modulation of WPI structure.

### Sample Viscosity after the Mixtures

Since there was no heating to promote denaturation, the change in viscosity occurred only due to the interaction between the surfactant and the protein. Pure WPI in water presented very low viscosity, similar to that in water, and could not be measured. WPI + CPC and WPI + SDS samples were visually similar viscous solutions, whereas the WPI + BC sample presented a high viscosity gel appearance and a lighter color. Figure 3(a) shows that WPI + CPC and WPI + SDS exhibited a Newtonian viscosity profile. WPI + CPC showed the highest viscosity throughout the speed range but was much more viscous than pure WPI. Figure 3(b) shows that WPI + BC had the highest viscosity and pseudoplastic behavior. These results suggest that the surfactants modified the protein and promoted some interactions even before the heat denaturation treatment.

**Figure 3:**
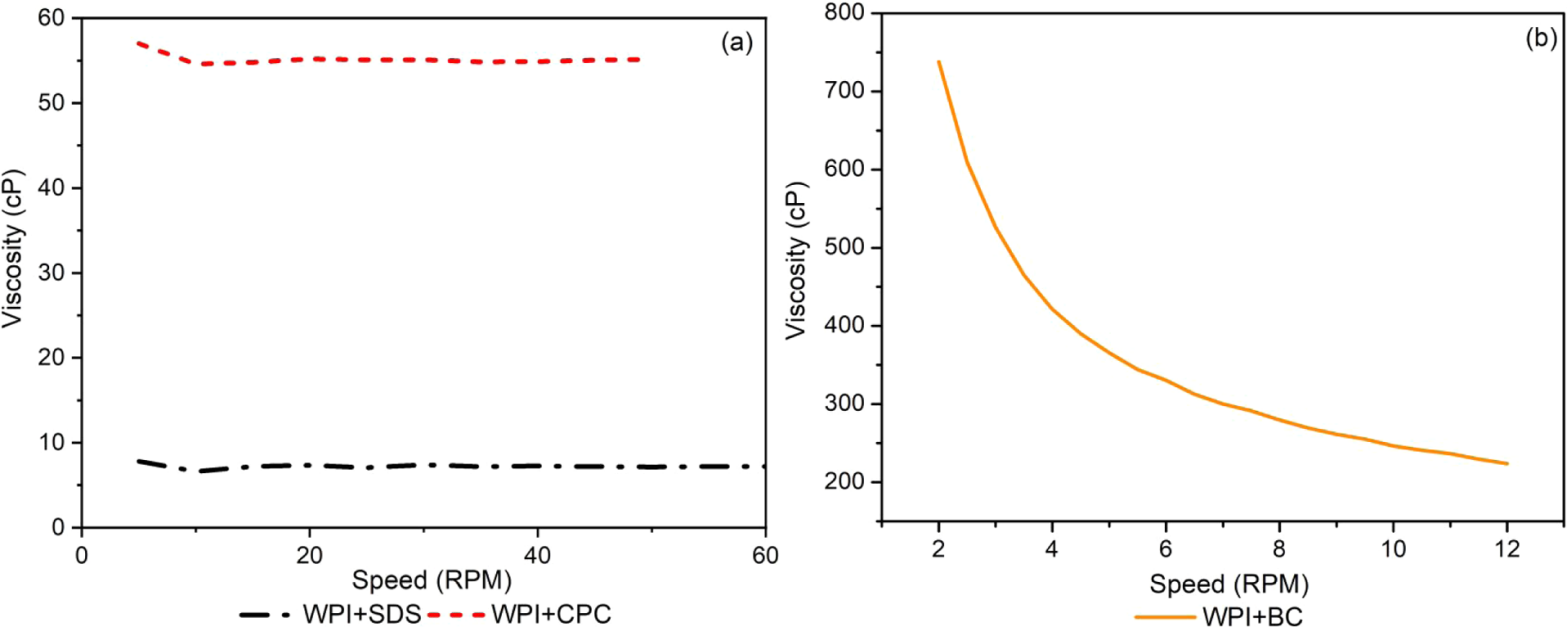
Viscosity graph for (a) WPI + SDS and WPI + CPC and (b) WPI + BC.

The increase in viscosity suggests complexation, formation of protein micelles, or even microstructuring by specific interactions (hydrophobic, electrostatic, π–π, etc.), especially in the WPI + BC system, suggesting the formation of more complex and intertwined structures such as protein–surfactant aggregates or physical networks that resist flow. This is consistent with the observed gel appearance and shear-thinning behavior. The low χ value for WPI + BC (χ = 0.0037) indicates high thermodynamic compatibility between the surfactant and the protein, favoring molecular miscibility and promoting stable interactions that increase the system’s viscosity.

The presence of hydrocarbon chains (in BC and CPC) may favor interpenetration in the hydrophobic regions of the proteins resulting in associative networks.^59,60^ Although SDS also presented χ <1, its higher value (χ = 0.6198) and strongly chaotropic character favor interactions that promote more denaturation and less ordered aggregation, which may result in lower viscosity if the aggregates do not form structured networks.^56^

### WPIT Characterization after Heating Denaturation

#### Dynamic Light Scattering (DLS)

DLS was performed for the samples after heat treatment and dilution. The particle size distribution is shown in Figure 4, and the Zeta potential values are summarized in Table 6. The combination of these parameters allows the assessment of both aggregate size heterogeneity and electrostatic stabilization. Analysis revealed highly polydisperse systems for all surfactant-treated samples, reinforcing the heterogeneity introduced by protein–surfactant interactions and thermal unfolding. The presence of bimodal size distributions highlights the formation of diverse aggregate populations.

**Figure 4:**
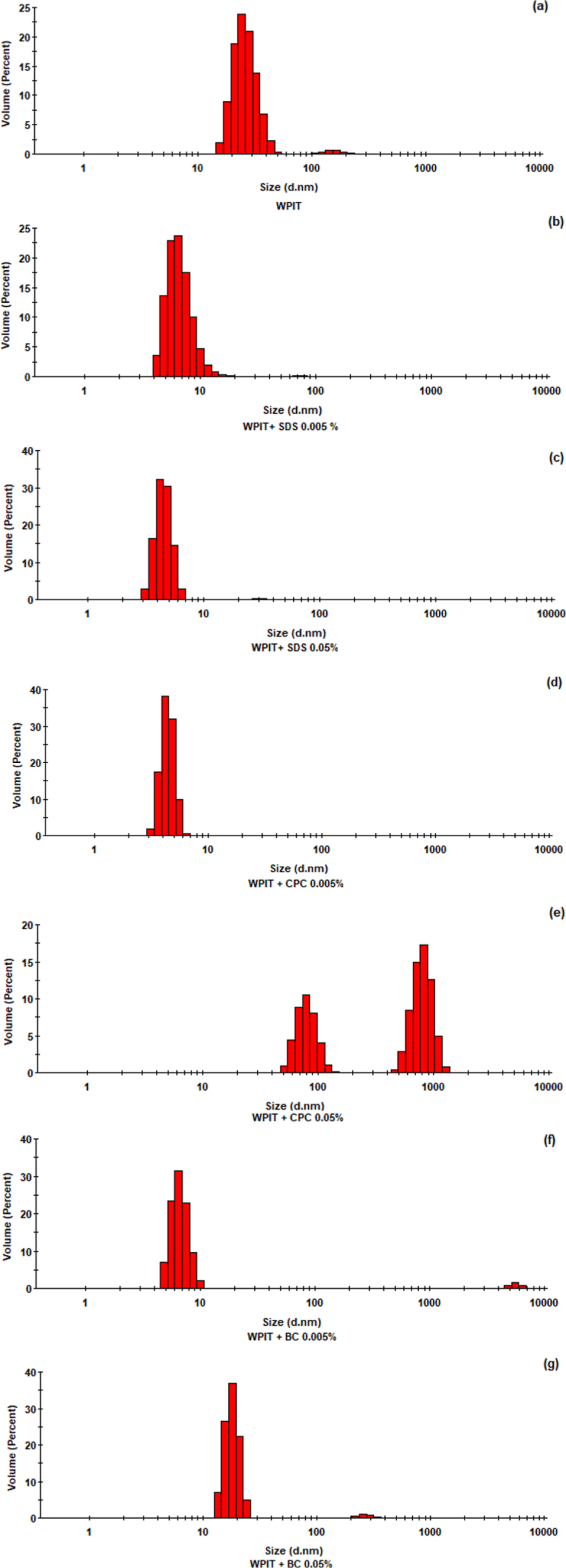
DLS size distribution profiles from samples of (a) WPIT, (b) WPIT + SDS 0.005%, (c) WPIT + SDS 0.05%, (d) WPIT + CPC 0.005%, (e) WPIT + CPC 0.05%, (f) WPIT + BC 0.005%, (g) WPIT + BC 0.05%.

**Table 6:** Zeta Potential of WPIT Samples

Sample	WPIT	WPIT + SDS 0.005%	WPIT + SDS 0.05%	WPIT + CPC 0.005%	WPIT + CPC 0.05%	WPIT + BC 0.005%	WPIT + BC 0.05%
Zeta (mV)	–13 ± 1	–13 ± 1	–15 ± 1	–9.8 ± 0.5	–10.0 ± 0.2	–13.1 ± 1.7	–11.5 ± 0.4

We observed notable alterations in the hydrodynamic diameter and zeta potential of particles, indicating the pronounced structural reorganization of WPIT in the presence of surfactants.

Figure 4(a) shows the DLS analysis of WPIT in water, revealing a major population around 20–40 nm, associated with intermediate-size protein aggregates formed during thermal denaturation, alongside a minor population near 100 nm, indicative of larger supramolecular assemblies. Heating promotes partial unfolding of proteins, exposing hydrophobic regions, and enhancing protein–protein interactions. The zeta potential of −13 mV, moderately negative, suggests some degree of electrostatic repulsion yet is insufficient to fully prevent the approach and clustering of proteins into larger aggregates. This behavior is typical of denatured proteins, as heat exposure enhances hydrophobic interactions through the exposure of buried domains.^61,62^

In the presence of a low SDS concentration (0.005%), Figure 4(b), the distribution shifted toward smaller sizes, with a predominance of particles in the 6–10 nm range and a marked reduction of larger aggregates. This effect can be attributed to the binding of the anionic surfactant to hydrophobic patches exposed during heating, which prevents protein–protein contacts. Although the zeta potential (−13 mV) remained similar to the control, surface charge saturation may not yet have been reached, while the solubilizing action of SDS was already evident, limiting aggregation.^56,57,63^

At a higher SDS concentration (0.05%), Figure 4(c), the distribution further narrowed, dominated by small, homogeneous particles, while the zeta potential became more negative (−15 mV), reflecting increased protein surface coverage and enhanced electrostatic repulsion. Under these conditions, SDS acts as a strong solubilizing agent, disrupting aggregates and maintaining proteins in smaller, soluble forms. This outcome is consistent with its chaotropic nature within the Hofmeister series, favoring protein destabilization and solubilization.^58,62,64^

For CPC at a low concentration (0.005%), Figure 4(d), the system exhibited a narrow distribution centered at 6–10 nm, indicating partial dissociation of aggregates into smaller subunits. This disaggregation can be explained by the initial binding of the cationic surfactant to negatively charged regions of the protein, reducing the self-association. The less negative zeta potential (−9.8 mV) suggests weaker electrostatic repulsion compared to the control but still sufficient to stabilize proteins as small particles.^59,60^

However, at a higher CPC concentration (0.05%), Figure 4(e), two distinct populations emerged, consistent with the formation of larger aggregates. This phenomenon can be attributed to extensive surface coverage by excess CPC, which reduces the availability of free charges and diminishes electrostatic repulsion, thereby promoting protein reorganization into larger clusters. This behavior aligns with the tendency of cationic salts in the Hofmeister series, which at low doses induce partial charge neutralization and disaggregation, but at higher concentrations drive reorganization and stabilization of larger aggregates.^15,17,59,60^

BC, due to its greater hydrophobicity, exhibited a distinct response. At a low concentration (0.005%), Figure 4(f), the distribution was broadened with the coexistence of aggregates of varying sizes, including a small fraction of very large particles. The zeta potential (−13.1 mV) remained close to the control, suggesting that hydrophobic interactions dominated over electrostatic forces, leading to heterogeneous assemblies.

In contrast, at a higher concentration (0.05%), Figure 4(g), the size distribution became more defined, dominated by smaller particles with only a residual peak of larger aggregates. This shift can be attributed to more efficient protein surface coverage by BC, which, despite reducing the zeta potential to −11.5 mV, limited direct protein–protein interactions and promoted a structural reorganization that decreased polydispersity. Thus, BC behavior reflects its strong hydrophobic character: initially inducing aggregation, but at higher concentrations stabilizing smaller aggregates through reorganization during heating.^6,12,65,66^

Overall, these results demonstrate that the response of WPI during thermal denaturation in the presence of surfactants is strongly dependent on their ionic nature and hydrophobicity, in agreement with the Hofmeister series trends. While SDS, an anionic chaotrope, primarily promotes solubilization and aggregation prevention, the cationic surfactants CPC and BC modulate colloidal stability through charge neutralization and strong hydrophobic interactions, inducing disaggregation at low concentrations but driving reorganization into larger aggregates under excess conditions.^13,33,37^

### Fourier Transform Infrared (FTIR) Spectroscopy

Fourier transform infrared (FTIR) spectroscopy assessed changes in the WPI secondary structure and intermolecular interactions after thermal denaturation and surfactant interaction. The analysis focused on the amide I region (1600–1700 cm^–1^), which arises primarily from C=O stretching vibrations in the peptide backbone and is highly sensitive to protein conformation. According to established spectral assignments, bands at 1615–1643 cm^–1^ and 1692–1697 cm^–1^ indicate β-sheet structures, 1647–1654 cm^–1^ correspond to disordered or random coil regions, 1651–1663 cm^–1^ to loop structures, 1653–1660 cm^–1^ to α-helices, and 1663–1695 cm^–1^ to turns or coils^67^ (Shivu et al. 2013). Spectral deconvolution of the amide I region using ATR-FTIR in thin films enabled the reliable quantification of these structural motifs without compromising protein integrity.

Figure 5 presents the FTIR spectra of native and treated samples, highlighting the amide I region where changes due to surfactant addition are evident when comparing WPIT (thermally treated WPI) to surfactant-modified samples. The amide I band is particularly informative in proteins with mixed secondary structures, as it appears as a broad, asymmetric envelope composed of overlapping sub-bands. Curve-fitting resolved and quantified the relative contributions of α-helix, β-sheet, turn, and random coil components.^49,68,69^

**Figure 5:**
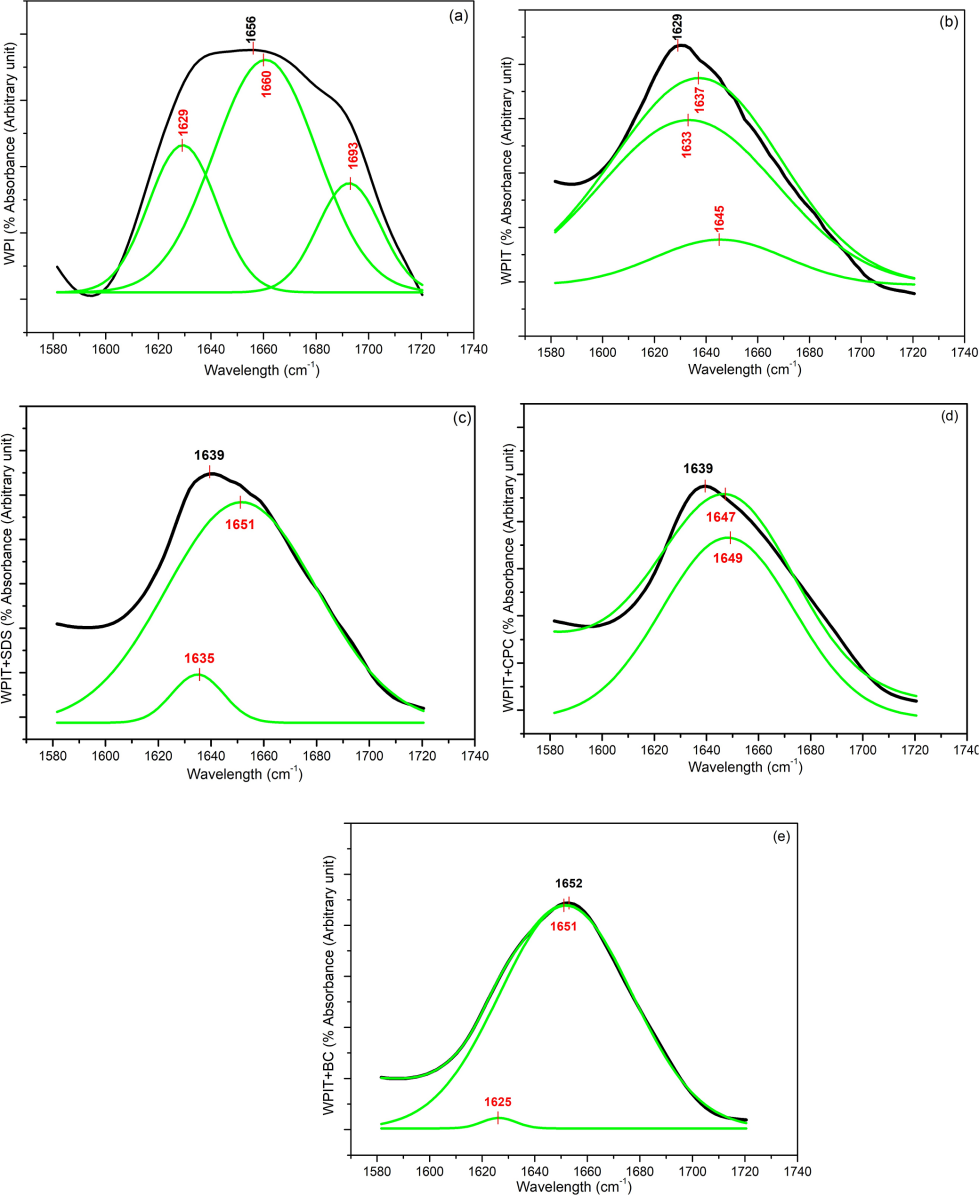
FTIR spectra in the 1800–1300 cm^–1^ within (a) WPI, (b) WPIT, (c) WPIT + SDS, (d) WPIT + CPC, (e) WPIT + BC.

Table 7 summarizes the deconvolution of the amide I band, highlighting the effects of thermal treatment and surfactant interaction on the secondary structure of the whey protein isolate (WPI). In native WPI, the spectral components were consistent with a globular protein structure characterized by prominent α-helix and β-sheet bands, reflecting the organized folding of the protein in its native state. After thermal treatment (WPIT), we observed a downshift in the main amide I band, accompanied by the emergence of new bands characteristic of aggregated β-sheet and disordered structures. This shift indicates significant conformational rearrangement and partial denaturation likely due to the exposure of hydrophobic residues and subsequent formation of intermolecular β-sheets—a hallmark of heat-induced structural transitions.^61,70–72^

**Table 7:** Deconvolution of the Amide I Band and Corresponding Protein Secondary Structures

Sample	Main amide I band (cm^–1^)	Assigned conformations	Deconvoluted bands (cm^–1^)	Assigned conformations^68–70^
WPI	1656	α- helix/loop	1629, 1660, 1693	β-sheet, α- helix/loop, turn
WPIT	1629	β-sheet	1633, 1637, 1645	β-sheet, β-sheet, random coil
WPIT + SDS	1639	β- sheet/random coil	1635, 1651	β-sheet, loop/random coil
WPIT + BC	1653	α- helix/loop	1626, 1651, 1654	β-sheet, loop, random coil
WPIT + CPC	1639	β- sheet/random coil	1647, 1649	random coil, random coil/loop

The presence of surfactants modulated these structural changes in distinct ways. In the WPIT + SDS sample, although β-sheet contributions remained, the spectra suggested a partial retention of ordered regions alongside an increase in the random coil content, pointing to a more flexible conformation. In this sample, the main band at 1639 cm^–1^ and deconvoluted peaks at 1635 and 1651 cm^–1^ pointed to a mixture of β-sheet and loop/random coil structures. This suggests that SDS, an anionic and strongly chaotropic surfactant in the Hofmeister series, disrupted native interactions but did not induce complete disorder. Instead, it appears to favor partial unfolding with the retention of some ordered motifs, likely due to selective binding along the polypeptide chain.

For WPIT + BC, the main peak at 1653 cm^–1^ and components at 1626, 1651, and 1654 cm^–1^ indicate a complex structural rearrangement. The presence of both β-sheet and α-helix/random coil contributions reflects a heterogeneous conformation, possibly due to BC’s cationic and amphiphilic nature. BC can engage in electrostatic interactions with acidic residues and hydrophobic interactions with nonpolar domains, which may stabilize certain regions while destabilizing others.

WPIT + CPC presented a main peak at 1639 cm^–1^, with deconvoluted bands at 1647 and 1649 cm^–1^, characteristic of random coil and loop structures. CPC, a cationic surfactant with a rigid aromatic headgroup, likely induces strong electrostatic and hydrophobic disruption of the native structure, favoring conformational flexibility and disordered arrangements over aggregation.

The spectral changes observed upon thermal treatment and subsequent surfactant addition reveal a clear trend: denaturation leads to β-sheet enrichment, and surfactants influence the extent and nature of this restructuring. SDS appears to maintain a partially ordered state, whereas BC and CPC promote structural heterogeneity characterized by disordered and flexible motifs, such as random coils and loops. FTIR analysis thus confirms that the WPI secondary structure is significantly altered by heat and further reshaped in specific patterns, depending on the surfactant involved.

### Circular Dichroism (CD) Spectroscopy

Circular dichroism (CD) spectroscopy complemented FTIR analysis and provided additional insights into the secondary structural transitions of WPI following thermal treatment and surfactant interaction. CD is particularly sensitive to conformational changes in proteins, especially in the far-UV region (190–250 nm), where the peptide backbone exhibits distinct dichroic signals.

α-Helical structures are typically identified by two characteristic negative minima near 208 and 222 nm, accompanied by a strong positive maximum around 190–193 nm. In turn, β-sheet structures generally display a single negative band between 215 and 218 nm and a positive peak close to 195 nm. Random coil or disordered conformations are characterized by a deep negative minimum around 195–200 nm, often lacking the dual minima associated with ordered secondary motifs.^73^

These spectral signatures enable both qualitative and semiquantitative assessment of protein folding states. When native WPI is compared to its thermally treated and surfactant-modified forms, shifts in CD signals serve as clear indicators of conformational rearrangement. The technique is thus highly effective in identifying unfolding events, structural loss, or stabilization induced by different surfactants.^74,75^

These spectral signatures enable both qualitative and semiquantitative assessments of protein secondary structure, particularly useful for comparing native and surfactant-modified conformations. Figure 6 presents the CD spectra of the samples, highlighting the effects of thermal treatment and surfactant addition on the WPI folding state. Changes in band positions and intensities across the spectra reflect distinct alterations in the α-helix, β-sheet, and random coil contents, supporting the structural transitions inferred from FTIR analysis.

**Figure 6:**
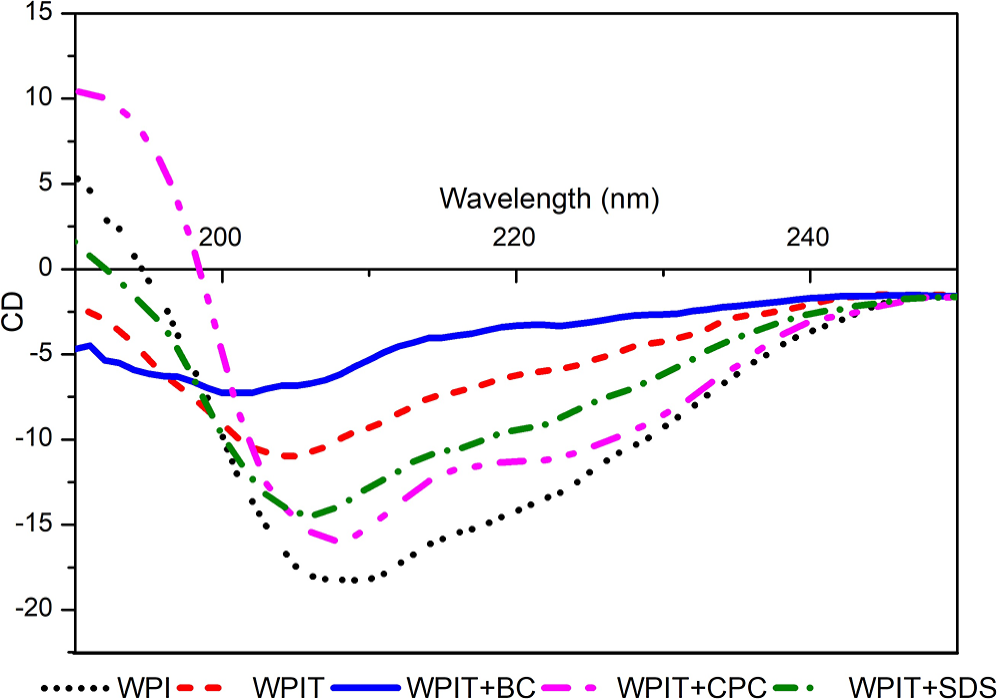
Circular dichroism analysis results.

Circular dichroism (CD) spectroscopy provided complementary insights into the secondary structure transitions of WPI, confirming and expanding upon the findings obtained by FTIR. Thermal treatment (WPIT) promoted a significant decrease in the intensity of the characteristic negative CD bands, accompanied by a spectral shift of the minimum toward approximately 205 nm. This pattern is consistent with the partial loss of the α-helix content and an increase in β-sheet and random coil conformations, as indicated by FTIR data. In FTIR, the main amide I band for WPIT shifted to 1629 cm^–1^ with deconvoluted bands at 1633, 1637, and 1645 cm^–1^, reflecting enhanced β-sheet structures and emerging disorder due to protein unfolding and aggregation.

Addition of surfactants further altered the secondary structure of the WPI in distinct ways. For WPIT + SDS, the CD spectrum retained a broad minimum near 208 nm, suggesting partial preservation of ordered elements. FTIR analysis also supported this interpretation, showing a main band at 1639 cm^–1^ and contributions from β-sheet and loop/random coil structures. SDS, an anionic surfactant with strong chaotropic character according to the Hofmeister series, disrupts hydrophobic domains and weakens protein folding; however, its selective interactions appear to allow partial structural retention, particularly for β-sheet elements.

WPIT + BC exhibited the lowest ellipticity in CD spectra characterized by a flattened curve and a loss of defined minima, indicating extensive conformational disruption and secondary structure loss. This is in agreement with FTIR results by a main band at 1653 cm^–1^ and deconvoluted peaks at 1626, 1651, and 1654 cm^–1^, pointing to a heterogeneous mix of β-sheet, loop, and random coil elements. BC, a cationic quaternary ammonium compound, is not classically positioned in the Hofmeister ion series but behaves similarly to destabilizing agents by promoting electrostatic and hydrophobic disruption, particularly with negatively charged residues such as Glu and Asp. Its strong interaction with exposed protein domains likely explains the pronounced unfolding observed. WPIT + CPC also showed a clear evidence of structural destabilization in the CD spectrum with a minimum shifted toward ∼200–202 nm, indicative of a predominance of random coil structures. FTIR results were in agreement, with a main band at 1639 cm^–1^ and deconvoluted peaks at 1647 and 1649 cm^–1^, confirming the shift toward disordered and flexible conformations.

Overall, CD and FTIR analyses reveal a coherent pattern of the secondary structure evolution. Thermal treatment alone induces a shift from α-helices to β-sheets and disordered structures. Surfactants modulate this response according to their chemical nature and interaction potential: SDS promotes partial unfolding with retention of order; BC promotes a highly heterogeneous and destabilized state; and CPC induces extensive unfolding and conformational flexibility. These effects align with predictions from the Hofmeister series, which help explain the varying degrees of protein destabilization observed, particularly the stronger impact of chaotropic cationic surfactants such as CPC and the intermediate behavior of SDS.

### Differential Scanning Calorimetry (DSC)

DSC tests comparatively evaluated the thermal events of the samples. Figure 7 shows the heating curves. Table 8 presents the values of the thermal events observed. The start of thermal event (T_onset_) observation provides a valuable approach to understanding the effects of molecular interactions within the studied system. A significant decrease in T_onset_ often reflects increased chain mobility as a consequence of the disruption of intermolecular interactions between chains.

**Figure 7:**
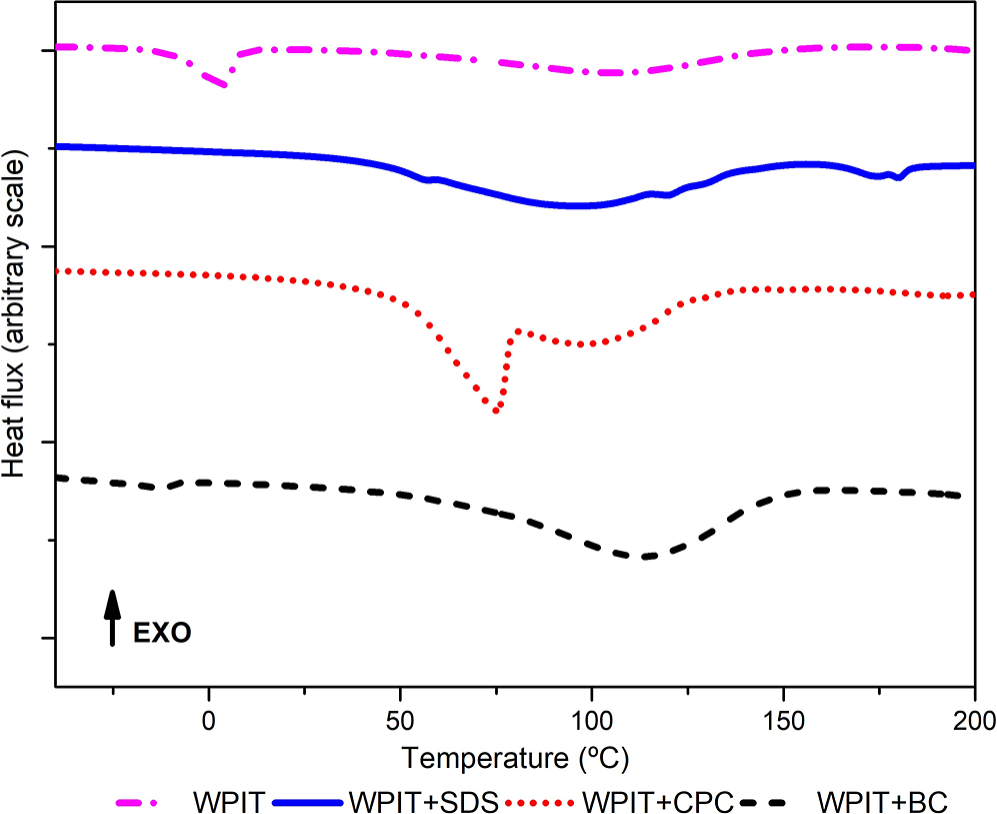
DSC heating curves of samples.

**Table 8:** Results of the Thermal Transitions of the Samples

Sample	T_onset_ ^a^(°C)	Peak (°C)	Δ*H* (J/g)
WPIT	–10; 57	3; 105	26; 0.7
WPIT + SDS	50; 116	56; 120	1; 1
WPIT + CPC	54; 81	75; 81	75; 46
WPIT + BC	–30; 63	–13; 113	6; 222

a Start of the thermal event.

WPIT presented one broad transition with a peak temperature around 105 °C. Tg of proteins without plasticizers is generally in the range of 120 to 250 °C, depending on the amino acid content.^76^ The samples were freeze-dried for good drying, but the TGA curves (shown in Figure 8) point out moisture residues, which cause the overlap of transition signals in this temperature range on the DSC curves. The same WPIT was analyzed by Cosate de Andrade et al. (2019),^77^ who found the same behavior. The authors also performed XRD analysis and observed that WPIT is amorphous, attributing the endothermic peak to water evaporation.

CPC exhibits a well-defined thermal transition at approximately 87 °C.^42^ Thus, one might expect that its incorporation into the protein system would result in a superimposed peak around this temperature, especially considering the relatively high CPC concentration in the formulation. However, the DSC curves suggest that thermal transitions in the WPIT + CPC system occur at slightly lower temperatures. This shift indicates that the observed peaks are not merely due to the melting of free CPC but are likely associated with structural reorganizations resulting from strong electrostatic interactions between CPC and the protein.

In turn, the sample containing zwitterionic surfactant BC exhibited the highest thermal transition temperature (113 °C) and the largest enthalpic response, suggesting the formation of highly ordered and thermally stable supramolecular structures. Interestingly, the formulation with SDS, although displaying low enthalpy values, showed thermal transitions at higher temperatures than those of the CPC-containing system. These differences indicate that the nature and strength of protein–surfactant interactions significantly influence the system’s thermal properties. Particularly, interactions that restrict the flexibility and mobility of the protein chains—such as those involving BC—tend to increase the material’s thermal stability.

CPC exhibits a thermal transition at around 87 °C, as reported in the literature. Therefore, its incorporation into the protein system could, in principle, lead to the appearance of a superimposed thermal event in this region, particularly considering its concentration in the formulation. However, in the DSC curves of the WPIT + CPC system, the broad endothermic events observed between approximately 80–120 °C occur at slightly lower temperatures and overlap significantly with the water evaporation/desorption region. This overlap prevents a clear assignment of these peaks to intrinsic CPC melting or protein phase transitions. Instead, these shifts are more conservatively interpreted as resulting from modifications in water–protein–surfactant interactions, which may alter the water-binding environment within the matrix rather than directly indicating well-defined structural reorganizations of the protein network.

Similarly, the WPIT + BC sample displayed the highest apparent transition temperature (≈113 °C) and the largest enthalpic response. However, given the strong contribution of moisture loss in this temperature range, this behavior is interpreted with caution, as it may reflect differences in water retention, distribution, or binding strength induced by the zwitterionic surfactant rather than the formation of highly ordered supramolecular structures.

The WPIT + SDS formulation, although showing lower overall enthalpy changes, exhibited endothermic events at higher apparent temperatures compared to those of the CPC system. This behavior is again attributed primarily to changes in water–matrix interactions and thermal moisture dynamics, influenced by the specific nature of protein–surfactant interactions.

Thus, rather than serving as direct evidence of enhanced thermal stability or structural transitions in the protein network, the DSC data are interpreted here as providing supportive information about changes in hydration behavior and thermal response of the systems. The assessment of thermal stability is therefore primarily based on the TGA results (Table 9), which more reliably reflect the degradation behavior of the materials.

**Table 9:** TGA Results

Sample	T_onset_ (°C)	Weight loss (%)	Residue (%)
WPIT	43	3	13
	299	74	
WPIT + BC	44	4	16
	209	40	
	319	16	
WPIT + CPC	49	5	16
	240	40	
WPIT + SDS	43	2	29
	235	23	
	316	16	

#### Thermogravimetric Analysis (TGA)

Thermal stability was analyzed by using TGA. Figure 8 shows the TGA curves (data were normalized from 100 °C to disregard moisture loss to compare the thermal stability of the samples). All numerical results are presented in Table 7.

**Figure 8:**
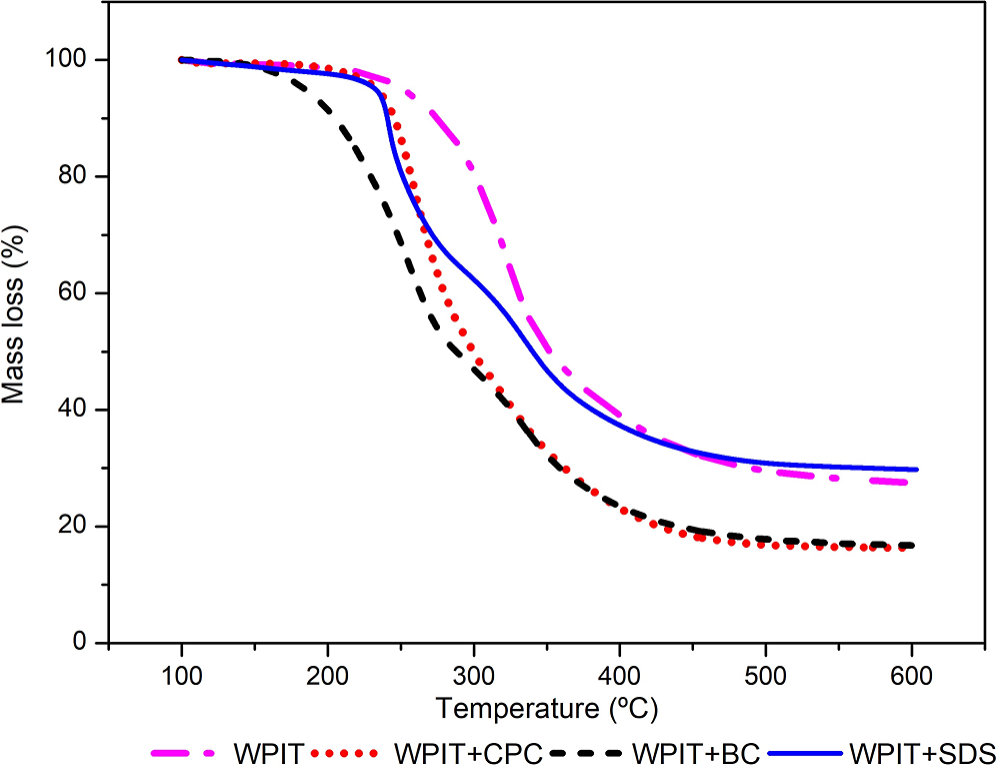
TGA curves for the samples.

The first stage of weight loss was observed below 100 °C, in which around 5 wt % was lost, corresponding to the removal of the free and bound water. The second and third stages of decomposition occur between 209 and 320 °C, primarily due to the breakage of covalent peptide bonds in the amino acid residues.^78,79^ All surfactants caused a decrease in the WPIT thermal stability, which reinforces the hypothesis that surfactants directly interfere with the stabilizing interactions of the protein. These results are consistent with data previously obtained by DLS, FTIR, CD, and DSC and support the hypothesis that surfactant addition combined with the thermal denaturation process favors protein structural reorganization.

Considering the prediction of compatibility by the Flory–Huggins theory, WPI + BC has the lowest χ value and the most severe reduction in thermal stability. The χ value suggests thermodynamic compatibility but does not necessarily correlate with preservation of intramolecular interaction. In WPI + BC, increased compatibility may facilitate deeper penetration of the surfactant into the protein matrix, enhancing disruption of stabilizing interactions and explaining the pronounced reduction in thermal stability observed by TGA.

### Rheology

Surfactant addition and the thermal denaturation process significantly altered the WPI rheological properties, reflecting important structural changes in the protein matrix. Figure 9 presents the complex viscosity (η*) curves for WPIT and its modified versions with different surfactants.

**Figure 9:**
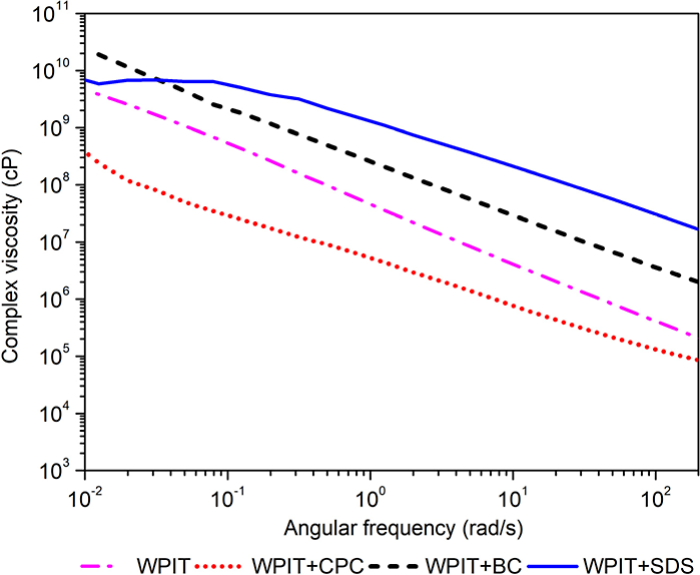
Complex viscosity (η*) of WPIT and the samples modified with surfactants.

It is important to emphasize that the rheological results discussed here refer to the complex viscosity (η*) of the thermally treated WPIT materials under the melt conditions. They do not refer to the apparent viscosity measured for the aqueous solutions prior to denaturation.

WPIT and WPIT + SDS samples showed higher complex viscosity values in the high-frequency region compared to the other formulations. At lower frequencies, WPIT + SDS exhibited a Newtonian plateau, as typically observed in thermoplastic-like polymeric systems, indicating stabilized chain mobility under melt conditions. In contrast, WPIT + CPC presented the lowest complex viscosity, suggesting that CPC incorporation compromises the formation of an interconnected 3D protein network after thermal processing. This behavior is attributed to a more extensive conformational disruption during denaturation, leading to increased chain mobility in the molten state.

WPIT + BC also showed a reduction in complex viscosity compared with WPIT. This result may appear counterintuitive when compared to the higher viscosity observed for the WPI + BC system in aqueous solution, which reflects hydrodynamic interactions and transient network formation under nondenaturing conditions. However, after thermal denaturation and film formation, the system undergoes a profound structural rearrangement, and the initial solution structuring induced by BC does not translate into a more resistant melt network. Instead, the strong interactions between BC and protein may facilitate deeper unfolding and network disruption, resulting in a more plasticized and less entangled structure and, consequently, lower complex viscosity in the molten state.

WPIT and WPIT + SDS samples showed higher complex viscosity values compared with the others for higher frequency regions. At lower frequencies, a Newtonian plateau is observed for WPIT + SDS as is normally observed for thermoplastic polymers. WPIT + CPC showed the lowest complex viscosity, suggesting that CPC modification reduced the protein’s three-dimensional network formation capacity, probably due to the induction of more profound conformational changes. WPIT + BC also showed a reduction in viscosity compared with WPIT.

Comparing the viscosity and the rheology results, we can point out: the solution viscosity should be interpreted as a descriptor of predenaturation structuring, whereas the melt rheology reflects the postdenaturation molecular organization and mobility. Direct comparison between these two parameters is thus intrinsically limited and must be treated with caution. The solution viscosity (before heating) reflects hydrodynamic interactions, electrostatic and hydrophobic associations, and transient network formation between partially folded protein molecules and surfactants in a fully hydrated environment. The complex viscosity (η*) measured after heating corresponds to the rheological behavior of the denatured, WPIT material under melt-like conditions, where proteins have undergone partial irreversible unfolding, aggregation, and reorganization into some new supramolecular network. Although WPI + BC showed the highest apparent viscosity in solution, this behavior does not necessarily translate to a stronger melt network. On the contrary, we now discuss that BC–protein interactions, while promoting stronger associative networks in solution, likely facilitate deeper penetration of the surfactant into the protein structure during heating. This process can intensify protein unfolding and disrupt stabilizing intra- and intermolecular interactions, resulting in a more plasticized and less interconnected network after thermal processing. Consequently, the melt-state WPIT + BC system exhibits a lower complex viscosity than WPIT.

Figure 10 presents the behavior of each sample in relation to *G*′ and *G*″. Comparison between the storage (*G*′) and loss (*G*″) moduli reveals that, for all samples, the elastic component (*G*′) predominated over the viscous component (*G*″) in most of the frequency range analyzed. This behavior indicates that despite WPI modification with surfactants and the thermal denaturation process, the protein matrix maintained a predominantly elastic characteristic, suggesting that some original intramolecular interactions were replaced by new stabilizing interactions. These new interactions, including hydrogen bonds and hydrophobic forces, can strengthen the bonds between protein molecules. As a result, both viscosity (η) and rigidity (*G*′) of the system tend to increase. However, heating can weaken hydrogen bonds, intensify electrostatic repulsions, and enhance hydrophobic interactions.^80–82^

**Figure 10:**
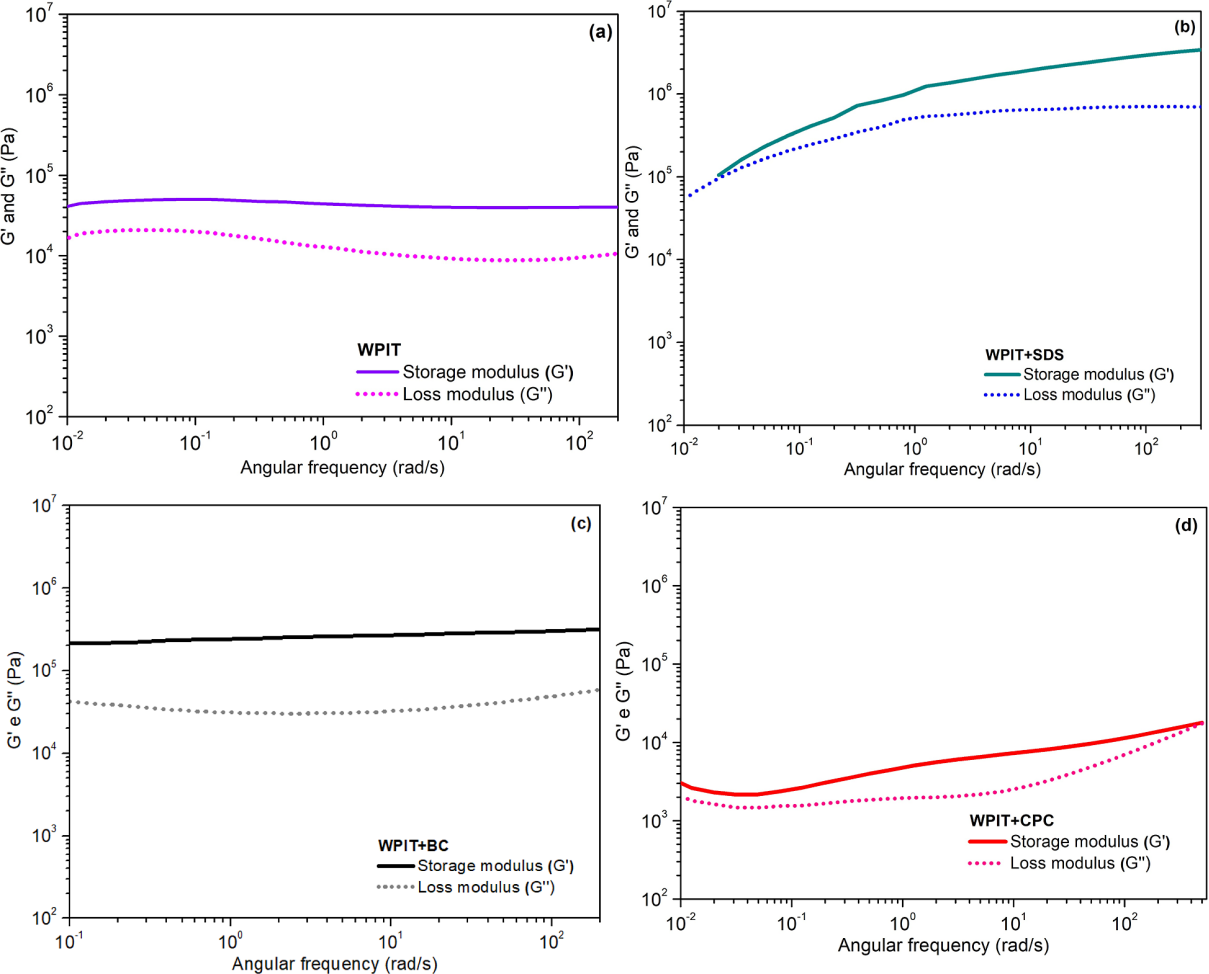
Behavior of *G*′ and *G*″ in (a) WPIT, (b) WPIT + SDS, (c) WPIT + BC, and (d) WPIT + CPC samples.

These results reinforce the idea that the type of surfactant and its electrochemical characteristics play a determining role in protein structure reorganization and, consequently, in defining the viscoelastic behavior of the modified protein.

However, this behavior is context-dependent. In the absence of heat, the observed effects stem purely from the surfactant–protein interactions with native protein conformations. When the system is subjected to thermal denaturation, the structural dynamics change considerably. Heating may cause the unfolding of WPI molecules, exposing hydrophobic regions and reactive groups that were previously buried. This conformational shift enhances the reactivity of proteins toward surfactants and may fundamentally alter the system’s rheological behavior.

## Conclusion

This study showed that structural modification by the surfactants cetylpyridinium chloride (CPC), benzalkonium chloride (BC), and sodium dodecyl sulfate (SDS) significantly alters the molecular conformation, thermal stability, and rheological behavior of WPI. Structural analyses (FTIR and circular dichroism) confirmed conformational rearrangements, particularly the transformation from α-helix to β-sheet structures induced by surfactant–protein interactions. Rheological characterization revealed that despite maintenance of elastic behavior after thermal treatment, the nature and intensity of the changes varied with the type of surfactant, indicating the formation of distinct intermolecular networks.

DLS and viscosity analyses supported the hypothesis that addition of surfactant exposes the hydrophobic regions and functional groups, facilitating protein reorganization into more processable structures. Thermal analyses (DSC and TGA) further evinced reductions in thermal stability, notably in the BC-modified samples, explained by Flory–Huggins predictions and underscoring the importance of considering Hofmeister-specific ion effects in protein–surfactant systems.

Overall, these findings highlight the strategic use of surfactants to tailor the WPI structural and physicochemical properties. They also point out the critical balance between protein denaturation and stabilization mechanisms necessary to optimize the material performance in biopolymer applications. However, other factors not addressed in detail here also exert a significant influence on protein–surfactant interactions and should be investigated further, such as the critical micelle concentration (CMC), a key parameter in determining the point at which surfactants begin to form micelles in solution.

## Supplementary Material



## Notes

Amplitude sweep rheological analysis defining the LVR (PDF)
