# Supplementary material for: Modification of Whey Protein Isolate with Surfactants Based on Hofmeister Series and Interaction Parameter
Source: ACS Omega. 2026 Feb 27;11(9):14600–15. doi: 10.1021/acsomega.5c10293 (PMC12980199; doi:10.1021/acsomega.5c10293)
Supplement: Supplementary file 1 [file ao5c10293_si_001.pdf]

Title: Modification of whey protein isolate with surfactants based on Hofmeister series and interaction parameter

Authors: Jhenifer Stefani Lopes(1); Marina Fernandes Cosate de Andrade(1); Ana Rita Morales(1)

Affiliation(1): Universidade Estadual de Campinas (UNICAMP), School of Chemical Engineering (FEQ), Department of Materials and Bioprocess Engineering (DEMBio), Av. Albert Einstein 500, Cidade Universitária, 13083-852 Campinas, SP, Brazil.

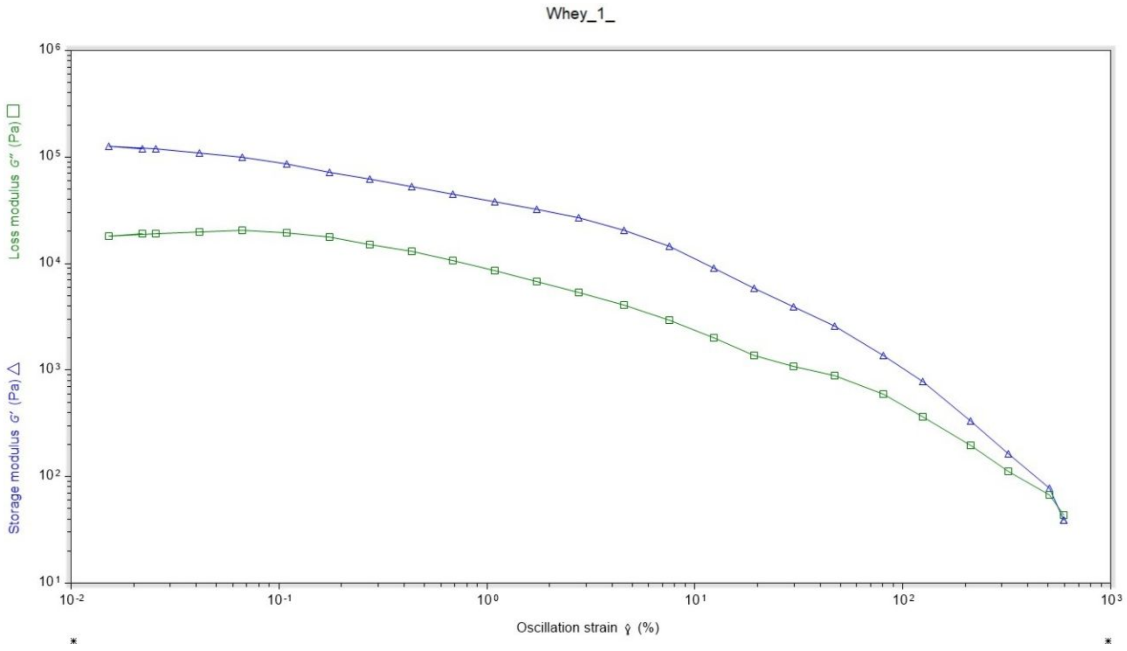

Figure S1. Storage (G′) and loss (G″) moduli as a function of strain amplitude obtained from amplitude sweep measurements, indicating the linear viscoelastic region.
